# Synergistic Effects of Nanosecond Pulsed Electric Fields Combined with Low Concentration of Gemcitabine on Human Oral Squamous Cell Carcinoma *In Vitro*

**DOI:** 10.1371/journal.pone.0043213

**Published:** 2012-08-23

**Authors:** Jing Wang, Jinsong Guo, Shan Wu, Hongqing Feng, Shujun Sun, Jie Pan, Jue Zhang, Stephen J. Beebe

**Affiliations:** 1 Department of Oral Medicine, School of Stomatology, Lanzhou University, Lanzhou Gansu, China; 2 College of Engineering, Peking University, Beijing, China; 3 Academy for Advanced Interdisciplinary Studies, Peking University, Beijing, China; 4 Department of General Dentistry, School of Stomatology, Peking University, Beijing, China; 5 Frank Reidy Research Center for Bioelectrics, Old Dominion University, Norfolk, Virginia, United States of America; Wayne State University, United States of America

## Abstract

Treatment of cancer often involves uses of multiple therapeutic strategies with different mechanisms of action. In this study we investigated combinations of nanosecond pulsed electric fields (nsPEF) with low concentrations of gemcitabine on human oral cancer cells. Cells (Cal-27) were treated with pulse parameters (20 pulses, 100 ns in duration, intensities of 10, 30 and 60 kV/cm) and then cultured in medium with 0.01 µg/ml gemcitabine. Proliferation, apoptosis/necrosis, invasion and morphology of those cells were examined using MTT, flow cytometry, clonogenics, transwell migration and TEM assay. Results show that combination treatments of gemcitabine and nsPEFs exhibited significant synergistic activities versus individual treatments for inhibiting oral cancer cell proliferation and inducing apoptosis and necrosis. However, there was no apparent synergism for cell invasion. By this we demonstrated synergistic inhibition of Cal-27 cells *in vitro* by nsPEFs and gemcitabine. Synergistic behavior indicates that these two treatments have different sites of action and combination treatment allows reduced doses of gemcitabine and lower nsPEF conditions, which may provide better treatment for patients than either treatment alone while reducing systemic toxicities.

## Introduction

Oral squamous cell carcinoma (OSCC) is the most widespread malignant oral cavity neoplasm [1], [2]. OSCC has a higher proportion of deaths than breast cancer and cervical cancer with 36,540 new cases and 7,880 deaths in the United States in 2010 [2]. Despite therapeutic advances using surgery, radiation, and chemotherapy, the 5-year survival rate has remained at 50–55% for the past four decades [2]–[4]. This disappointing outcome strongly suggests that we needed to improve treatments of OSCC.

Presently, chemotherapy is one of the most important treatment methods for malignancy. However, misuse and overuse of drugs could induce adverse effects and chemotherapeutic drug resistances are common [5]–[8]. Therefore, avoiding drug resistances and adverse effects of chemotherapy treatment in cancer and improving therapeutic outcomes have recently gained considerable attention. One way to enhance uptake of chemotherapeutic agents is by electroporation therapy (EPT), which has been more recently referred to as electrochemotherapy (ECT); EPT would include gene electrotransfer (GET) [9] and irreversible electroporation (IRE) [10], both of which are used for cancer therapy. The primary biological effect of conventional electroporation is by reversible permeabilization of target cell plasma membranes. Short (millisecond, ms or microsecond, µs), relatively low voltage, electrical pulses can create micropores in plasma membranes, allowing entrance of poorly permeating agents such as macromolecules, proteins, drugs or genes [11]–[16]. Electrochemotherapy has been considered an interesting alternative in treatments of head and neck cancer [17], [18]. However, ECT only increases bioavailability of membrane impermeable drugs by permeabilizing plasma membranes [19].

Unlike conventional electroporation, nanosecond pulsed electric fields (nsPEFs) exhibit extremely short pulse durations, high voltage, but low energy and non-thermal effects [20]. They create large transmembrane potentials across membranes [21] and nanopores in plasma membranes as well as in intracellular membranes [22]–[25]. Recently, treatment with nsPEFs is emerging as a novel stimulus for inducing tumor cell death. Apoptosis can be induced by nsPEFs in various cancer cell lines *in vitro* [26]–[30], and in B16f10 melanoma tumors [32] and in Hepa l–6 hepatocellular carcinoma *in vivo* [33]. Current studies show that nsPEFs can induce several cellular responses including calcium bursts from the endoplasmic reticulum [34]–[37], DNA fragmentation [29], and caspase activation [26], [30], [38]. However, there are currently no studies on tumor cell treatments with nsPEFs in combination with chemotherapy agents.

In this study, a deoxycytidine analog drug gemcitabine (2′, 2′-difluorodeoxycytidine) is employed in combination with nsPEFs. Gemcitabine has shown activity in a variety of solid tumors, including breast, head and neck, bladder, ovary, lung, and pancreas [39]–[41]. Current studies suggest primary antitumor mechanisms of gemcitabine include reduction of apoptotic thresholds, interference with DNA replication and blockade of DNA synthesis [42]–[44]. Huang *et al.* [45] reported antitumor activity of gemcitabine increased when calcium concentrations increased. Since nsPEFs induce formation of nanopores in plasma membranes [25], these pulses do not enhance delivery of drugs to cells like that observed in ECT. However, our study provides evidence that nsPEFs with low doses of gemcitabine have a strong synergistic effect on cell death in OSCC. This has potentially important clinical relevance given toxicity for gemcitabine when used as a radiosensitizer for treatment of head and neck carcinomas [46] as well as high burden of anemia, thrombocytopenia and other associated untoward effects [47]. These observations are demonstrated using MTT viability assays, clonogenic assays, flow cytometry, scanning electron microscopy and transwell invasion assays. Synergistic effects with combinations of gemcitabine and nsPEFs are observed in all of these assays except cell invasion, which exhibited additive effects. The demonstration of synergy indicates that these two therapies have different sites of action that coordinately enhance OSCC cell death by apoptosis and necrosis.

## Materials and Methods

### Cell line and cell culture

The cell line used in this study was Cal-27 (ATCC American Type Culture Collection CRL-2095), a human squamous cell carcinoma cell line of the tongue. Cal-27 cells were cultured in DMEM medium, supplemented with 10% dialyzed fetal calf serum and 2 mM glutamine (Invitrogen). No antibiotics were used. Cells were cultured as monolayers and maintained in exponential growth in a humidified air atmosphere with 5% CO_2_/95% at 37°C. Cal-27 cells were harvested at 80–90% confluence by treatment with 0.25*%* trypsin/0.53 mM EDTA solution and prepared for *in vitro* experiments.

### Application of nanosecond pulsed electric fields (nsPEFs)

**Figure 1:**
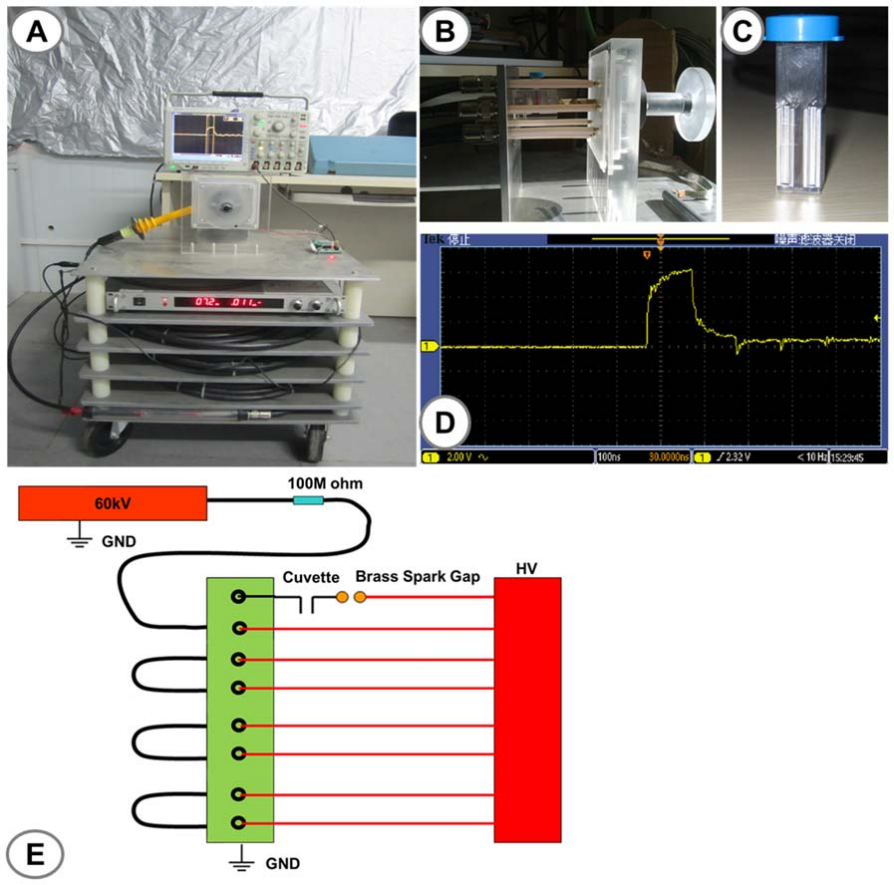
A schematic diagram of experimental setup for nsPEFs on Cal-27 cells. A schematic diagram of experimental setup for nsPEFs on Cal-27 cells. A) NsPEF generator; B) Pulse excitation region; C) 0.2 cm gap cuvette (Biosmith); D) The typical waveforms of nsPEFs; E) Circuit design for nsPEFs.

In this study, we used a nanosecond pulsed electric field generator as previously described with a duration of 100-ns [48]. Electric fields were varied from 10 kV/cm to 60 kV/cm. Waveforms were monitored using a digital phosphor oscilloscope (DPO4054. Tektronix.USA) equipped with a high voltage probe (P6015A.Tektronix.USA). The pulse power devise is shown in Fig. 1. Cal-27 cells were harvested and resuspended in cell culture media with a concentration of 2.0×10^6^ cells/ml. A 500 µl cell suspension (1×10^6^ cells) was placed in 0.2 cm gap cuvette (Biosmith, aluminum plate electrodes) and exposed to nsPEFs. To explore possible synergistic effects of nsPEFs combined with low concentrations of gemcitabine on Cal-27 cells *in vitro*, 20 pulses with 100 ns durations with electric field of 10 kV/cm, 30 kV/cm, 60 kV/cm, and a gemcitabine concentration of 0.01 µg/ml were applied alone and in combination with Cal-27 cells and then treated with nsPEFs. According to different treatments, cells were divided into four groups. Group A was the control group and received neither gemcitabine nor nsPEF. Group B was treated with gemcitabine, group C was treated with nsPEF (10 kV/cm, 30 kV/cm or 60 kV/cm). Group D was exposed to nsPEF as in group C plus gemcitabine.

### Cell proliferation test

Anti-proliferative effects of gemcitabine on Cal-27 cells were determined with the 3-(4, 5-dimethylthiazol-2-yl)-2, 5-diphenyltetrazolium bromide (MTT) dye uptake method. In this study we seeded 8×10^3^ cells/well into 96-well flat bottom (Costar) plates. When cells began to grow exponentially, they were treated with gemcitabine and desired concentrations (0.01, 0.1, 1.0, 10, 100 µg/ml). After incubation for 24, 48, and 72 h, 20 µl MTT (5 mg/ml) was added to each well, and cells were further incubated at 37°C for 4 h. The medium was then removed and 200 µl of DMSO was added to dissolve the reduced formazan product. Dye intensity was then read on a micro plate reader (Bio-Rad) at 492 nm.

Using this same method, we investigated proliferation effects of nsPEF combined with gemcitabine. Cal-27 cells were harvested and resuspended with a concentration of 2.0×10^6^ cells/ml. A 500 µl cell suspension (1×10^6^ cells) was placed in 0.2 cm gap cuvette (Biosmith) and exposed to nsPEFs. After treatment group D cells were treated with nsPEFs and then incubated with 0.01 µg/ml gemcitabine. Then cell proliferation was analyzed after 24, 48, and 72 h by MTT assay.

### Clonogenic assay

Clonogenic regrowth efficiency was determined by plating single cell suspensions in medium onto the bottom of cell culture dishes. Cal-27 cells in control and treated groups were exposed to different nsPEF intensity of 0 kV/cm, 10 kV/cm, 30 kV/cm, and 60 kV/cm, then cells were incubated in 37°C for 6 hours to allow attachment to the plastic bottom before the medium was replaced with or without gemcitabine. The control group and gemcitabine group had 200 cells seeded and other treated groups had 2000 cells seeded in 60 mm plate. After incubation for 10 days, cell colonies were fixed and stained with 0.1% crystal violet. Colonies (≥50 cells) were counted for computing percent growth inhibition.

### Cell apoptosis evaluation by flow cytometry

A Annexin V-FITC Apoptosis Detection Kit (BD Biosciences Pharmingen) was used for assessing apoptosis induced by nsPEF combined with low concentration of gemcitabine. Annexin V-FITC and propidium iodide (PI) were used to evaluate normal cells (no staining), early apoptotic cells (annexin positive, PI negative) and necrotic cells (annexin and PI positive). After treatment with nsPEFs, groups B and D were incubated in fresh media with 0.01 µg/ml gemcitabine. All cell groups were incubated 2 h at 37°C. Cells were then collected, stained with Annexin V-FITC in a dark at room temperature for 15 min, and then stained with PI on ice for 30 min. Samples were assessed by FACSauto flow cytometry (Becton Dickinson, USA).

### TEM observation for morphological changes

Morphological changes were observed by transmission electron microscopy (TEM). Cells were first fixed with 2.5% glutaraldehyde/2% osmium tetroxide for no more than 20 minutes and stained with uranyl acetate and lead citrate.

### Synergism quotient calculation

The synergism quotient is calculated by subtracting baseline values from all treatments and then dividing effects of combined treatments by the sum of individual treatments. A synergism quotient greater than 1.0 indicates that there is synergism for a given measured response.

### Cell invasion assay

Cell invasion was assessed by a modified Boyden assay using transwell chambers (Costar, Cambridge, MA) with 8 µm pore polycarbonate filters that were coated with 50 µg/ml of Matrigel™ (BD, Biosciences, Bedford, MA) diluted in serum-free medium. Cal-27 cells were treated with nsPEFs (10 kV/cm, 30 kV/cm, 60 kV/cm) plus gemcitabine. Cells (2.0×10^4^ cells/chamber) were seeded in the top of cylindrical cell culture inserts in DMEM media plus 2.5% FBS. DMEM media with 10% FBS was placed in wells below and cells were allowed to migrate through the filter for 48 h at 37°C in 5% CO_2_. Non-migrating cells were removed from upper surfaces of chambers by scrubbing with a cotton swab. Migrated cells on the lower membrane were fixed in 100% methanol and stained with 0.1% Crystal Violet (Invitrogen) for 20 min at 4°C. Invasion cells were counted under a microscope.

### Statistics

Data were analyzed using one-way ANOVA, post hoc, Bonferroni and Dunnett's test for analyses of multiple group comparisons. Statistical analyses and graphics were performed using the program SPSS version 16.0. Results were expressed as the mean±SEM. In all cases P<0.05 was taken as the level of significance.

## Results

### Synergistic suppression of proliferation with nsPEFs and gemcitabine

**Figure 2:**
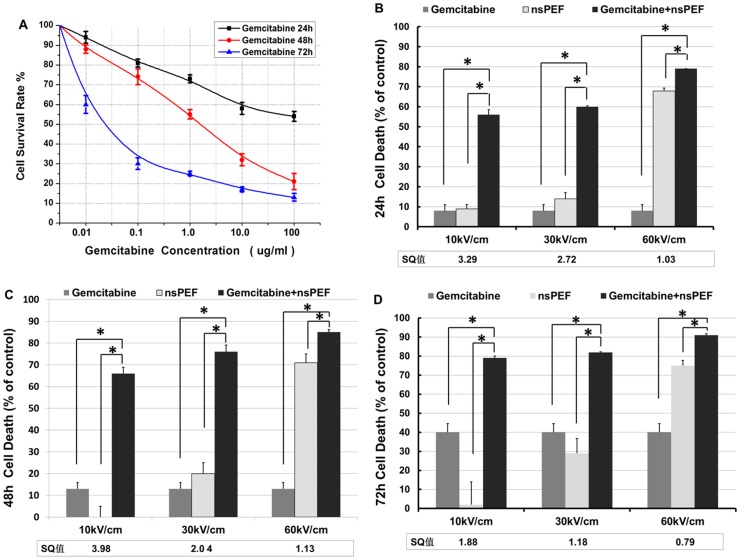
NsPEF combined with gemcitabine synergistically inhibit growth in Cal-27 cells. Inhibition of cell proliferation was assessed by MTT assay. A) Cal-27 cells were treated with various concentrations of gemcitabine for 24 h, 48 h, 72 h. B, C, D): Cell death rates of Cal-27 cells were determined after combination treatment for 24 h, 48 h and 72 h. Results are presented as the percentage of the decreased values from the control cells. Insets in B) and C) show synergism quotients at every electric field with gemcitabine combination. The synergism quotient is defined in Materials and methods. The results presented are averages of three independent experiments each done in triplicate and expressed as the mean ±SEM. *p<0.01, one way ANOVA with Bonferroni/Dunnett's test compared to the nsPEF group and gemcitabine group.

Effects of gemcitabine on proliferation of Cal-27 were first determined using MTT assay. As indicated in Figure 2A, results showed a clear concentration- and time-dependent inhibitory effect of gemcitabine on Cal-27 cell survival. When a concentration of 2.3 µg/ml of gemcitabine was applied, Cal-27 cell viability was reduced to a level of 50% of control samples at 48 h. Based on these results, we chose 0.01 µg/ml gemcitabine as an extremely low concentration for combination treatments. Figures 2B, C and D show effects of gemcitabine and nsPEFs (10, 30 and 60 kV/cm) alone and in combination in an MTT cell death assay 24, 48 and 72 hours after treatment. When used alone, there was an electric field-dependent increase in nsPEF-induced cell death. Combination groups showed a more significant inhibition than the sum of effects of nsPEF and gemcitabine alone, especially at 10 kV/cm and 30 kV/cm, where synergism quotients were >3 and 2 times better than the sum of each treatment alone. In addition, results showed that combination treatments have an electric field strength- and time-dependent effect on Cal-27cell proliferation. However, as effects of nsPEFs become greater, the ability to see synergism decreases because as effects of electric fields alone approach a maximum response, the “window” for seeing synergism is progressive decreased; when nsPEFs produce a maximal response, the ability to see synergism is lost.

### Electric field-dependent effects of nsPEFs and synergism on Cal 27 cell survival

**Figure 3:**
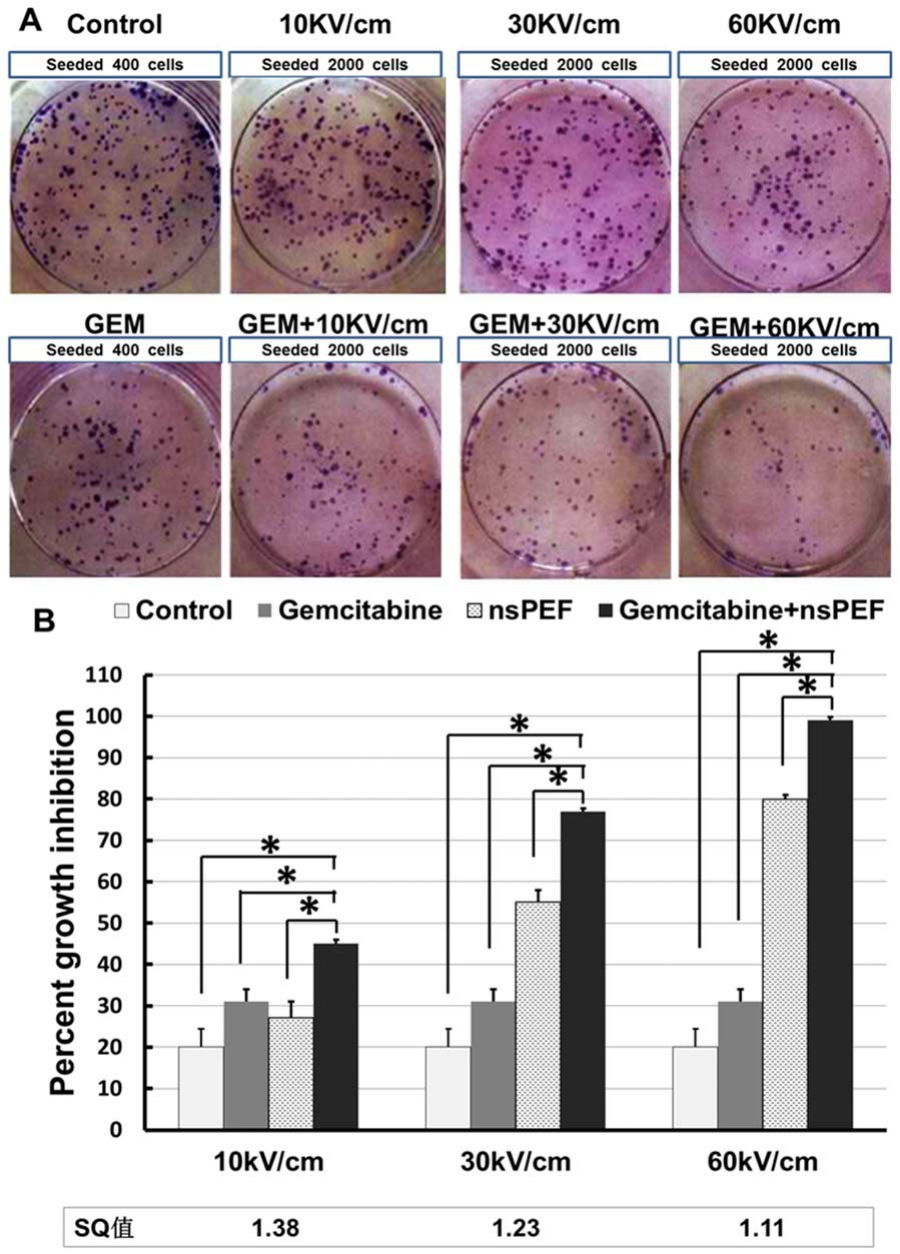
Effect of the combination of the nsPEFs and gemcitabine in the colony formation of Cal-27 cells. A) Photographic difference in colony formation in treated groups. B) Gemcitabine (0.01 ug/ml) in combination with nsPEFs at 10, 30 and 60 kV/cm, respectively. Data are expressed as percentages of growth inhibition in reference to growth of untreated control cells. The synergism quotient is defined in Materials and methods. The results presented are average of three experiments each done in triplicate and expressed as the mean ±SEM. *p<0.01, one way ANOVA with post-hoc Bonferroni/Dunnett's test compared to the control group, nsPEF group and gemcitabine group.

To study effects of treatment effects on Cal-27 cell growth, cells were treated with nsPEFs and gemcitabine, and cell viability was assessed by clonogenic assays (Figure 3). Again, there was an electric field-dependent increase in growth inhibition with nsPEF treatment. The combination treatment resulted in inhibition of colony formation of Cal-27 cells when compared with the sum of the nsPEF group and gemcitabine group alone However, in this application synergism was less than in proliferation assays. This is due to *in vitro* conditions that allows drug to be present with continued action during entire incubation times. This is unlike an *in vivo* situation where the drug would have limited time-action effects. Nevertheless, results from clonogenic assays were consistent with MTT data as shown in Figure 2, suggesting that nsPEFs combined with low concentrations of gemcitabine significantly inhibited cell growth in Cal-27 cells.

### Synergistic effects of nsPEFs and gemcitabine on apoptosis and necrosis

**Figure 4:**
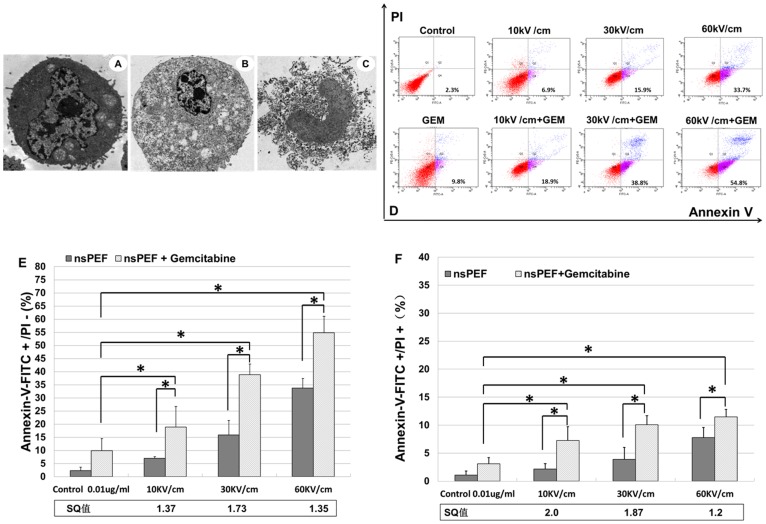
NsPEFs combined with gemcitabine significantly induced Cal-27 cell apoptotic death. A) The graph shows normal Cal-27 cells, which have intact plasma membranes and nuclear envelopes; B) nsPEF combined with gemcitabine induced apoptosis of Cal-27 cells. From the graph, blebbing membranes are clear; C) nsPEF plus gemcitabine induce necrosis in Cal-27 cells, which have ruptured nuclear and plasma membranes; D) Characterization of apoptosis after PI and Annexin V-FITC staining; E, F) Synergism of percent cells showing apoptosis and necrosis, respectively, is determined by treatment of cells with nsPEF and gemcitabine alone and in combination. The results presented are average of three experiments each done in triplicate and expressed as the mean ±SEM. *p<0.01, one way ANOVA with post-hoc Bonferroni/Dunnett's test compared to the control group, nsPEF group and gemcitabine group.

Numbers of apoptotic and necrotic cells were determined by annexin V-FITC and PI double staining. Greater numbers of cells showing early apoptosis were observed when nsPEFs were combined with the low concentration of gemcitabine. Early apoptosis (PS externalization without PI staining) detection results by flow cytometry were shown in Figure 4D, and statistical data were shown in Table 1. Results show that in combination groups, PS externalization was induced markedly at 10, 30 and 60 kV/cm as indicated by synergism quotients greater than 1.0. In addition, combinations of nsPEFs with gemcitabine also exhibited synergistic actions on necrosis (annexin-V-FITC and PI double staining); synergism quotients for necrotic cells were also greater than 1.0 at all electric fields tested. Notice that there was a “response window” for observing optimal synergism for both apoptosis and necrosis. This is typical, because as individual effects become too great, the “response window” for synergism becomes smaller and eventually no synergism can be seen due to effects of one treatment.

**Table 1:** Effect of nsPEFs combined with Gemcitabine on Cal-27 apoptosis.

Group	Annexin-V-FITC+/PI−(%)	P-values	SQ	Annexin-V-FITC+/PI+(%)	*P-values*	SQ
Control	2.3±0.6	---	---	1.1±0.7	---	---
Gemcitabine	9.8±4.6	P = 0.016	---	3.1±1.1	P = 0.013	---
10 kV/cm	6.9±0.3	P = 0.001	---	2.2±0.9	P = 0.011	---
30 kV/cm	15.9±5.5	P = 0.041	---	3.9±1.1	P = 0.007	---
60 kV/cm	33.7±3.6	P = 0.03	---	7.8±1.8	P = 0.009	---
Gemcitabine+10 kV/cm	18.9±7.1	P = 0.047	1.37	7.3±2.4	P = 0.024	2.00
Gemcitabine+30 kV/cm	38.8±4.1	P = 0.003	1.73	10.1±1.6	P = 0.003	1.87
Gemcitabine+60 kV/cm	54.8±6.2	P = 0.004	1.35	11.5±1.3	P = 0.001	1.20

Synergism of apoptosis rate was determined by treatment of cells with nsPEF and Gemcitabine in combination. The apoptosis rate of nsPEF-Gemcitabine combination divided by the sum of single control, nsPEF, Gemcitabine groups to obtain the values of synergism quotient. P-values stand for statistical significance compare the treated samples with control samples.

The presence of apoptotic and necrotic cells was also evident upon a morphological analysis. Morphological characteristics of normal cells by electron microscopy are: uniform distribution of cytoplasm and integral cellular membrane (Figure 4A). Typical morphological changes of Cal-27 cells after combination treatment were observed through transmission electron microscope. After combination treatment for 4 hours with nsPEFs plus 0.01 µg/ml gemcitabine, Cal-27 cells exhibited morphological characteristics of apoptosis including nuclear condensation, oversize cytoplasmic particles and vacuoles as well as smooth, integral cellular membrane and intact organelles (Figure 4B). In addition, characteristics of necrosis, including karyopycnosis, endolysis, damaged organelles, diffused chromatin, and ruptured plasma membrane were observed in Cal-27 cells after combination treatment (Figure 4C).

### Absence of synergism of nsPEFs and gemcitabine on cell invasion

**Figure 5:**
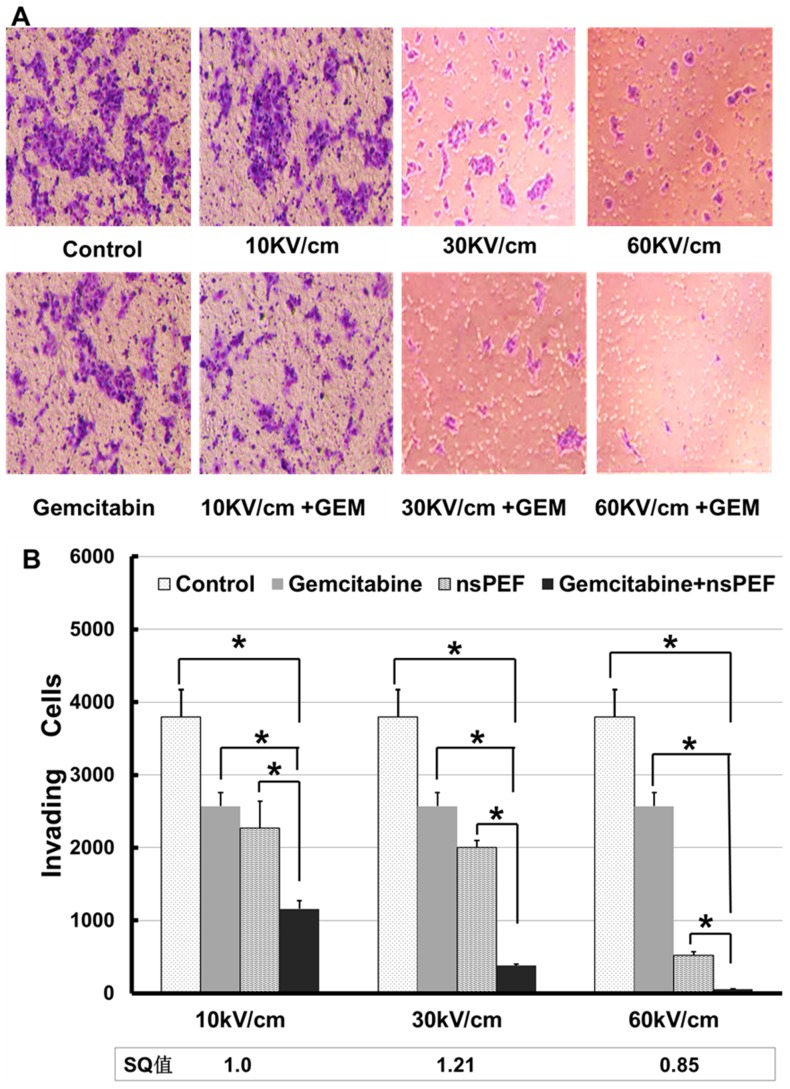
Invasion assay by nsPEF and gemcitabine combination treatment. A) The representative microscopic fields of invasion cells on the bottom of transwell inserts. B) Quantitation of cell invasion assay by counting invaded cells. Five microscopic fields were arbitrarily chosen and the average invaded cell number was determined. The results presented are average of three experiments each done in triplicate and expressed as the mean ±SEM. *p<0.01 one way ANOVA with post-hoc Bonferroni/Dunnett's test compared to the control group, nsPEF group and gemcitabine group.

Invasion assays were performed as described in Materials and methods. As shown in Figure 5, both gemcitabine and nsPEFs inhibited invasion potentials of Cal-27 cells. In addition, inhibitory effects of nsPEF on invasion were electric field-dependent. When the potential for synergism was analyzed from effects of the combination of gemcitabine and nsPEFs, there was no synergistic effect on Cal-27 cell invasion.

## Discussion

Applications of nsPEF are emerging as a novel stimulus for tumor treatment [26]–[33]. Electric fields interact with plasma membranes, intracellular organelles [20] and alter cell functions such as mobilizing calcium [34]–[37], dissipating mitochondria membrane potentials (ΔΨm) [27], [49]–[50], and damaging DNA [29], [51] as well as inducing apoptosis [26]–[30], [49] and other forms of cell death [27], [49], [52]. Although applications of nsPEFs are effective to eliminate melanoma [31], [32] and hepatocellular carcinoma [33] in mice *in vivo,* understanding underlying mechanisms require further analysis. Gemcitabine is used to treat several cancers, including head and neck tumors. However, like most cancer therapies, multiple treatments are needed either in combination or in sequence. Even then based on present outcomes, more efficacious treatments are needed. In this study, we investigated effects of nsPEFs combined with a low concentration of gemcitabine on proliferation/survival, apoptosis/necrosis and invasion in Cal-27 human OSCC cells *in vitro*. We hypothesized that by combining gemcitabine with nsPEFs, the concentration of gemcitabine could be reduced significantly to include efficacy without significant side effects. By analyzing a number of effects, we found synergistic activity with treatment combinations to inhibit proliferation and survival and induce cell death by apoptosis and necrosis, but not to affect cell migration/invasion. Results also show that combination treatments inhibit Cal-27 cell proliferation in a time- and strength-dependent manner. Although results for proliferation inhibition and colony formation were in agreement with each other, synergism for colony formation showed less synergism because effects of individual treatments were quite effective alone. This suggests that even lower gemcitabine concentrations and lower electric fields could be effective.

These *in vitro* results suggest that for treatments of Cal-27 cells, and likely other gemcitabine-sensitive tumors, in combination with nsPEFs treatments, gemcitabine concentrations can minimize drug side effects. Since nsPEFs have minimal side effects [32]–[33], it should be possible to effectively eliminate tumors with combination treatment with minimal untoward effects. Since these synergistic actions could be therapeutically advantageous, it will be important to advance these *in vitro* studies to an animal model and determine whether different treatment scheduling can enhance synergism.

The presence of synergism suggests that mechanisms of action of each treatment are likely at different sites and/or through different pathways. Gemcitabine interferes with replication and synthesis of DNA and exhibits self-potentiation [42]–[44]. However, gemcitabine-induced apoptosis appears to be cell type-specific [54]. Gemcitabine actions are complex and it may have different cell death mechanisms in different cell types. The same thing can be said of nsPEFs.

Exact sites of action for nsPEF-induced cell death are still in question. DNA is a possible site [29], [32], [51]. Gemcitabine and nsPEFs could act at different sites on DNA like that observed with gemcitabine and ionizing radiation [46]. It has also been suggested that nanopore formation is a major cause of apoptosis [22]. The presence of nanopores in plasma membranes depolarizes cell membrane potentials [24], [25]. Subsequent fluxes of the ubiquitous second messenger calcium, which regulates myriad cell responses, can upset homeostatic mechanisms. Suspected nanopore formation in inner mitochondria membrane causes dissipation of ΔΨm [27], [49], [50] and elevated levels of intracellular calcium could exacerbate this by overloading and upsetting mitochondria calcium homeostasis. This can disrupt a wide range of functions for maintenance of life as well as induction of death. Thus, cell membrane nanopores can threaten life and promote death.

Regardless of sites of action, this study provides accumulating evidence that nsPEFs induce cell death through multiple pathways including apoptosis (caspase-dependent) and necroptosis/necrosis (caspase-independent) [27], [49], [52]. Gemcitabine/nsPEF combinations exhibited synergy for both types of cell death, again suggesting actions at different sites. Because apoptosis and necrosis can both affect mitochondria [53], these organelles are likely primary sites for both cell death mechanisms. Since ATP production is needed for apoptosis but not necrosis, ATP levels could determine which type of cell death is induced [54]. Like gemcitabine, actions of nsPEFs are complex. Since this is the first study to investigate uses for nsPEFs and a chemotherapeutic agent, additional work will be required to determine mechanisms of each agent alone before mechanisms for their synergistic effects can be determined.

Synergism observed with gemcitabine and nsPEFs is essentially the same as dose enhancement effects observed with gemcitabine as a radiosensitization agent. Concentrations and exposure times for gemcitabine here and elsewhere were similar to those used in radiosensitization [55]. However, variations of treatment schedules and intervals were not investigated in our study and these factors may be important like that observed for radiosensitization [55], [56]. Since nsPEFs were used before gemcitabine in this study, it is possible that nsPEFs sensitizes Cal-27 cells to gemcitabine. It will be useful to study possibilities for synergistic effects using even lower concentrations of gemcitabine and lower electric fields with variable treatment order, times and intervals.

Combinations of gemcitabine with nsPEFs on invasion exhibited additive effects, but not synergism. Nevertheless, both treatments were potent invasion inhibitors. Since cellular mechanisms for cell motility and invasion versus proliferation and cell death are much different, synergistic effects of these treatment combinations show some selectivity for actions on cellular mechanisms.

It is again noted that the treatment approach used here with gemcitabine is distinct from uses of chemotherapeutic agents in electrochemotherapy (ECT), which only increase plasma membranes permeability of poorly permeable chemotherapeutic drugs. In contrast, gemcitabine is readily membrane permeable and both it and nsPEFs have their own sites and mechanisms of action. It is most likely that these two therapies act at different sites or pathways significantly diminishing side effects yet providing cooperative actions that inhibit proliferation and lead to tumor cell death by apoptosis and necrosis.
